# A Model to Support Fluid Transitions between Environments for Mobile Augmented Reality Applications

**DOI:** 10.3390/s19194254

**Published:** 2019-09-30

**Authors:** Tiago Davi Oliveira de Araújo, Carlos Gustavo Resque dos Santos, Rodrigo Santos do Amor Divino Lima, Bianchi Serique Meiguins

**Affiliations:** Computer Science Postgraduate Program, Federal University of Pará, Belém 66075-110, Brazil

**Keywords:** adaptive model, mobile augmented reality, indoor, outdoor

## Abstract

The adaptability between different environments remains a challenge for Mobile Augmented Reality (MAR). If not done seamlessly, such transitions may cause discontinuities in navigation, consequently disorienting users and undermining the acceptance of this technology. The transition between environments is hard because there are currently no localization techniques that work well in any place: sensor-based applications can be harmed by obstacles that hamper sensor communication (e.g., GPS) and by infrastructure limitations (e.g., Wi-Fi), and image-based applications can be affected by lighting conditions that impair computer vision techniques. Hence, this paper presents an adaptive model to perform transitions between different types of environments for MAR applications. The model has a hybrid approach, choosing the best combination of long-range sensors, short-range sensors, and computer vision techniques to perform fluid transitions between environments that mitigate problems in location, orientation, and registration. To assess the model, we developed a MAR application and conducted a navigation test with volunteers to validate transitions between outdoor and indoor environments, followed by a short interview. The results show that the transitions were well succeeded, since the application self-adapted to the studied environments, seamlessly changing sensors when needed.

## 1. Introduction

The Augmented Reality (AR) technology awakened the interest of the people as early as the 1970s, with a promise of a new interface that would link digital content and the real world. This interest helped to keep the AR research active, even when the peripherals used to make the AR application were uncomfortable, had poor quality, and little or no mobility. [1].

The advent of mobile devices has given mobility to the AR, enabling users to adopt the technology in more places than just on their desk, with the limitation of reduced screen size. However, even with all the advances, researchers still seek improvements to allow the use of AR technology in everyday life of people, avoiding the stigma of being considered as just a gadget [2,3,4].

Although the advances in software and hardware have boosted AR, there are still significant challenges to overcome, aiming to make it massively popular and productive. Azuma [5] indicated the lack of more accurate tracking techniques to improve the quality of the registry (the act of aligning digital information with the real scene), both for internal and external environments, also considering changes in the weather and day time. Azuma further emphasized the lack of adaptive AR applications which are responsive to environmental and user movement changes.

A significant challenge is the lack of precision in the registry [6], mainly when the user is indoors and the application uses the Global Positioning System (GPS) as primary localization technology to perform the tracking. The problem occurs because GPS sensors have low accuracy on mobile devices [7], especially when there are obstructions between the device and the satellites that calculate position. The low accuracy directly affects the registry quality, and, consequently, the user experience.

Proposals of technologies to mitigate the problem of GPS accuracy can be divided into two broad groups: continuous tracking and non-continuous tracking. Continuous tracking technologies include WIFI, Bluetooth, and magnetic fingerprint. For most proposals in this category, it is necessary to create a map with fixed reference points, such as an access point or beacon, to infer the user location. Non-Continuous tracking technologies include computer vision techniques that can recognize natural or artificial markers that point in the direction that users should go to find their point of interest. Trackers in this category provide less navigation information to the user, making the application less reactive because it depends on user interactions with the markers. Both tracking groups are generally geared towards a specific type of environment and are therefore limited in generalization.

This paper presents an adaptive model to perform transitions between environments for MAR (Mobile Augmented Reality) applications. The model contains a manager module that receives data from the real world and automatically selects the best tracking techniques between available long-range sensors (e.g., GPS), short-range sensors (e.g., Wi-Fi and Bluetooth), and computer vision tracking, to adapt the application to changes in the real world. In addition, the model assumes the locations are a tree-based structure: each location is a node (e.g., a building), with the locations inside it being children nodes (e.g., the rooms). This hierarchy positions the user at the root node and drills-down to the most precise location, where the most suitable sensor is chosen. For instance, the model identifies the country, state, city, neighborhood, building, floor, and room where the user is. Each level has a corresponding most suitable sensor, and, when the user transits through nodes, the application changes them accordingly.

The rest of this paper organize as follows. Section 2 reviews the related works. Section 3 presents the proposed adaptive model. Section 4 introduces the prototype developed to validate the model. Section 5 describes and discusses the evaluation performed in the prototype. Section 6 concludes and draws future works.

## 2. Related Works

The first work that dealt with indoor/outdoor transitions did it with notebooks, Head-Mounted Displays (HMD), and extra cameras, instead of actual mobile devices, presenting some solutions to this area in a pre-smartphone era [8]. The authors proposed a video-game application with a low-cost architecture that achieved moderately accurate tracking. It used GPS and fiducial markers for positioning and highlighted the hardships of registration errors when indoors and close to buildings.

Piekarski et al. [9] also used GPS and fiducial markers for indoor and outdoor positioning, and their follow-up work [10] partially improves the transition between indoor and outdoor environments by using more than one camera within the user’s device and placing markers on transition places. The limitation of this approach is that the user must carry equipment around, and the fiducial markers have to be big and distributed around the place. Following the HMD trend, the work of Siegl et al. [11] uses inertial sensors and image recognition to add information to objects in the scene based on user interaction. However, their system is not applied to outdoor environments.

For mobile devices, Yu et al. [12] developed a MAR application that has modules for indoor and outdoor Point of Interest (POI) navigation. It uses GPS and image recognition separately depending on the ambient type but lacks an answer for a seamless indoor/outdoor navigation transition. The Mirror World [13] deploys a heterogeneous AR solution for a multiplayer game where the transition is done by “AR bubbles”: pre-defined locations that trigger a switch between AR and VR environments when the user passes through them.

In the work of Campbell et al. [14], a scenario of an HMD with no access to sensors is used as proof of concept for the SIXTH middleware. This middleware allows the usage of external sensors outside of the user’s device for an AR application, and, while it has features to integrate various sensors, it does not tackle any particular solution for transitions, such as indoor/outdoor scenarios.

The work of Ruta et al. [15] proposes a mobile navigation assistant application and framework that has an AR interface. This system uses map annotations and semantic description for Location-Based Services and POIs, and use this information allied with Wi-Fi trilateration to infer user location indoors. Their framework can be used for both indoor and outdoor environments, but the validation was only done indoors.

As a general solution for mobility, the work of Liu et al. [16] presents an approach of user mobility for system reusability and portability. This approach considers different technologies, mainly for indoor positioning, to deliver for the user a system that can seamlessly help the user in a use case of transitional navigation. As for AR, it shows visual cues for path routing, but does not present a computer vision solution.

Chen et al. [17] proposed a system for a wide-area localization using vision techniques combined with information obtained from GPS and inertial sensors. Additionally, they argued that the use of vision-based techniques is still a challenge on smartphones due to the high processing and memory demands.

Polvi et al. [18] proposed an AR application that uses vision-based algorithms among them the Simultaneous Localization And Mapping (SLAM) for the AR registry. Hashemifar et al. [19] proposed the hybrid use of SLAM combined with the strength of Wi-Fi signals for indoor use.

Table 1 shows an overview of the related works in chronological order, with the technologies they have used and how they were combined. Although using a variety of localization methods, few works have shown a strategy for adapting the use of each sensor to the context of the environment and those proposing applications with fluid transitions have not used all kinds of localization methods.

There are many works with hybrid approaches (combined use of various types of localization techniques), although the transition from using them fluidly in the same application has been briefly addressed in the literature. Therefore, this paper aims to propose a model and a validation prototype for a seamless transition between environments (e.g., outdoor/indoor transitions). The related research served as the basis for choosing the technologies suitable for each environment context.

## 3. Proposed Architectural Model

The adaptations of MAR applications to the environmental context should occur gradually and transparently for the user. Hence, such applications should infer the location of the user through the information that the mobile device can have at that moment, or classify scenes and objects of the environment through captured images. In addition, user position can be divided hierarchically, from the broadest position (e.g., which country, city, and region the user is in) to a more precise and restricted positioning (e.g., what building, floor, and room the user is in). We decided to classify places where the positioning is an area as AOI (Area of Interest) and places such as buildings as POI (Point of Interest). Finally, we call IOI (Item of Interest) any object that is in a POI (Point of Interest) and has associated information.

### 3.1. AOI, POI, and IOI

The model relies on a classification of the location into three types that we named AOI, POI, and IOI. It is necessary to classify the location of the user to decide the strategy for environmental adaptation transitions.

**Figure 1:**
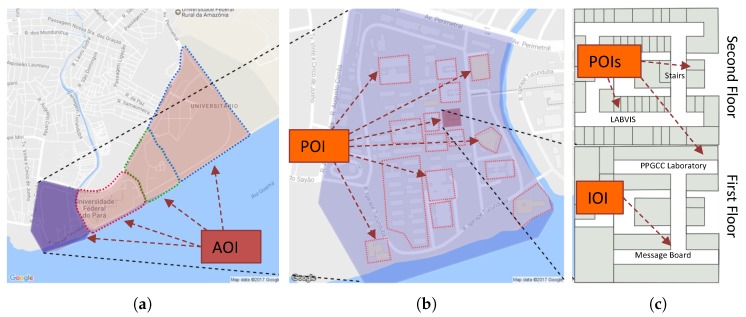
(**a**) Two levels of AOIs are illustrated; (**b**) the blue AOI of (**a**) with its internal POIs is illustrated; and (**c**) the lower and upper floors of the red POI shown in (**b**).

Figure 1 illustrates the hierarchical representation of AOIs, POIs, and IOIs on the map and floor plans, performing a drill-down into the hierarchy from left to right. Figure 1a shows a set of AOIs, Figure 1b shows the previously selected AOI with internal POIs, and Figure 1c shows the floor plan of the selected POI. In this case, the building has two floors, requiring an internal localization strategy to define both the floor and the location.

#### 3.1.1. AOI

AOI are geographic areas (circles, rectangles or polygons) and may contain other children AOIs or POIs. The model has to classify the user position based firstly on the root AOIs in the hierarchy. The AOIs localization can be long-range sensors, such as GPS.

The definition of AOIs with the obtained user position makes it possible to check which AOI the user is in, searching for the lowest AOI level in the hierarchy. Once discovered in which AOI the user is and at what hierarchy depth, the application can self-adapt by updating the content with the adequate level of detail, for instance modifying the representation of the virtual icons (e.g., size, shape, and aggregation), presenting all children and siblings of the current AOI to the user, and if necessary using computer vision algorithms for nearby POIs.

#### 3.1.2. POI

POI are locales that can contain other POIs inside and can have a short-range localization method. For instance, a building can be a POI and can have other POIs such as floors, corridors, and rooms. The first POI level (i.e., a child of an AOI) represents an example of a transition between outdoor and indoor environments.

**Figure 2:**
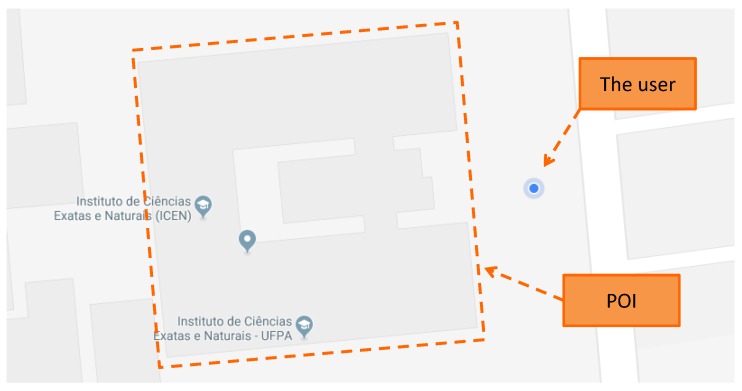
The user is near a POI; this moment represents the beginning of the transition between environments.

When the user is near to a POI (inside an AOI), the system can use short-range localization methods to confirm whether the user is inside the POI. Figure 2 illustrates the moment when the user is near to a POI, and the transition begins to occur.

#### 3.1.3. IOI

IOI are items that the user can interact with, such as paintings, books, and any other object of interest. The IOIs can be inside a POI or AOI, and the user cannot enter inside an IOI.

### 3.2. The Model

**Figure 3:**
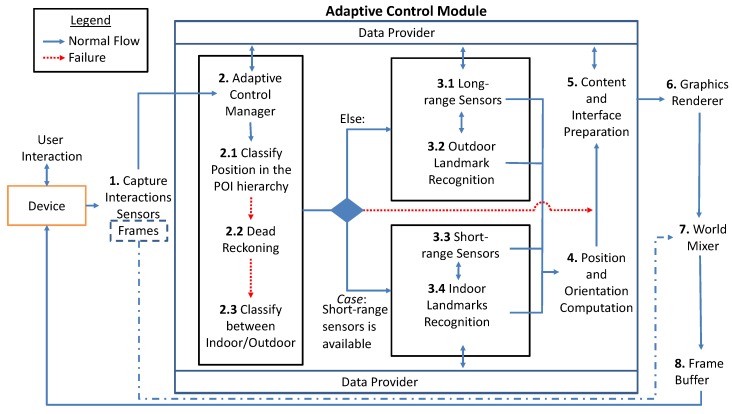
The architectural model is adaptive to user transitions between environments.

Figure 3 shows the adaptive model in detail. The model is based on the traditional AR pipeline (capture, register, and render) and decides the most appropriate strategies for locating, registering, and presenting content according to each environment accordingly to the user location.

The first task of the Adaptive Control Module is to receive and analyze data obtained from the sensors and the camera of the mobile device (1). An analysis for the Adaptive Control Manager (2) is done to classify if the user location is in an AOI or POI using long-range sensors (2.1). There are three possible situations for the location: outside of any AOI, within an AOI, or a within POI.

When reading the positioning sensors, errors and problems can occur, such as the loss of signal caused by object blocking. If the initial verification fails, then the adaptive model uses dead reckoning (2.2) for a while, which is the algorithm of predictive analysis that uses previous measurements to calculate a direction and velocity vector, estimating the user position when the information is not available [22,23,24]. However, the dead reckoning suffers from the cumulative errors, since previous measurements are used to estimate a new position. If there is a miscalculation in any measurement, the next measurements build upon the wrong data.

If the dead reckoning is already running for a certain amount of time, such that the cumulative error has become too large, computer vision techniques are used (additionally) to classify if the image captured by the device camera is an indoor or outdoor environment (2.3), confirming the user position.

Once the adaptive model classified the user position hierarchically within AOIs and POI, the model starts a method for calculating a more accurate location of the device. If the classification is AOI and the accuracy is adequate, the data of this approach are used (3.2). Otherwise, the adaptive model applies computer vision algorithms (3.1), which can be calculated in the cloud or by the device itself. A composition of the two approaches is also possible (long-range sensors plus computer vision).

If the classification is a POI, short-range sensor (3.3) is used, such as Wi-Fi networks, Bluetooth beacons, and RFID (Radio Frequency IDentification). Otherwise, computer vision algorithms are used (3.4) to predict user position, and it is also possible to combine the two approaches. Additionally, the camera pose should be calculated for the correct visualization of the augmented content (4), usually using the inertial sensors of the mobile device and computer vision algorithms.

The adaptive model verifies (considering location, distance, and orientation criteria) which AOIs and POIs’ contents are available for visualization. The content of the AOIs or POIs next to the user is obtained through a request to a content provider (5). Icons of AOIs can represent aggregated POIs if the user is outside the referred AOI, making the POIs that are within that AOI represented by the AOI icon, reducing the visual clutter on the screen.

The transition between environments can be adapted based on a POI area (when there is a good GPS accuracy) or using computer vision algorithms to classify the environments. If the GPS is not accurate, indoor/outdoor scene recognition algorithms [25,26,27,28] can be used to decide whether the user is indoors or not, or in which place the user are.

If a failure occurs in the process of locating the mobile device, the application should display a feedback message to the user. Depending on the type of failure, the application may request information that corrects it or may only display a notification.

After that, the traditional AR pipeline continues, the AOIs and visible POIs are rendered (6), and the system forwards the result to the Digital Mixer (7), which records the virtual content with the scene captured by the camera. After merging these two contents, the augmented image becomes available in the frame buffer (8) of the device.

When the icons are already on display, the user can interact with virtual content in the augmented environment. This action aims to search for content associated with the selected AOI or POI.

For MAR applications, the usage of various sensors can hinder the user with heavy usage of battery [29]. Thus, transition sequences between indoor and outdoor must address this problem appropriately. The coordinated exchange between GPS and Wi-Fi sensors can bring down the power usage of the device using the proposed model, allowing transparent power management. Turning off the GPS as the user enters an indoor environment reduces power usage and, knowing the points near the exit, the model can turn it on again without losing any position information. Even with a weak signal, Wi-Fi usage can be used efficiently over 3G or 2G networks to reduce power usage [30], thus, alongside the GPS, the power usage can be brought down by not using these connections. The model reduces power consumption as a positive side effect of transition states management.

The user positioning is essential for augmented reality since it enables the adequate alignment of the augmented content, thus the accuracy of positioning has a direct impact on the registration quality. Hence, there is a need to decide the best positioning strategy during the user movement to generate a more accurate registry. The novelty of this model is to intelligently decide which sensor is more appropriate depending on which level of hierarchy (AOI or POI) the user is. This strategy can improve the registration since the best available sensor is selected, and can also save resources (e.g., power, processing, and memory).

## 4. Prototype Application

A MAR application was developed to test and validate the proposed model. The application uses several technologies available in mobile devices, such as inertial sensors, camera, GPS, Wi-Fi, and Internet access; it also makes use of computer vision algorithms for pattern recognition in images, especially when there is low precision or failure in the sensors.

The application adjusts itself to the different environments by performing transitions between sensors. Mainly, if the user is outdoors, then the application displays information about the surroundings and registers the virtual elements appropriately, for example, using GPS and inertial sensors. Likewise, when the user is indoors, the application self-adjusts to show the internal contents of the current building. Since GPS usually has low accuracy indoors, the application should automatically change the tracking strategy to an internal positioning or a computer vision strategy.

### 4.1. Contents of Test

**Figure 4:**
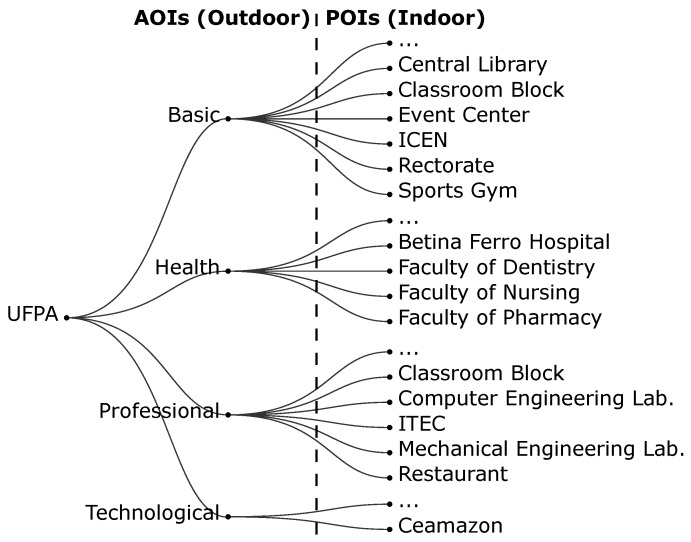
The hierarchical organization used in the model evaluation.

The prototype has content about the Federal University of Pará (UFPA), including the organization of AOIs and POIs in a hierarchical structure. Figure 4 shows the hierarchical structure present in the prototype: the first level is the UFPA, followed by its sectors (Basic, Professional, Health, and Technological) and by the POIs, which represent the buildings within the university.

The ICEN (Institute of Exact and Natural Sciences), located in the Basic sector of UFPA, has indoor localization to test the prototype adaptation. The ICEN has two floors, and its Wi-Fi antennas are located only on the second floor. In addition, the ICEN has FC-2 classroom (second floor), LABVIS laboratory (second floor), and PPGCC laboratory (first floor), which are part of the experiments.

As the application is still in the prototype phase, this content is embedded in the application. However, the idea of the presented model is that the application requests this content from a content provider depending on the user’s current location. For instance, once the application has located the user in the Basic sector of UFPA, then it asks: Which buildings are around? Which of these buildings contains content (e.g., media, short-range location sensors, and image landmarks)? The application makes these requests to the server, providing the current location, and the server delivers the content for that location. Thus, the application can scale more easily, since new content entries are centralized on the server and the user does not need to have all content on their smartphone; the application only downloads the content that is relevant to the moment on-demand from the server.

### 4.2. Graphical User Interface

The prototype application has an AR browser that adapts the GUI and the content according to user transitions between AOIs and POIs. Additionally, it has textual search functions of POIs, routes to a POI, and visualization of POI contents.

**Figure 5:**
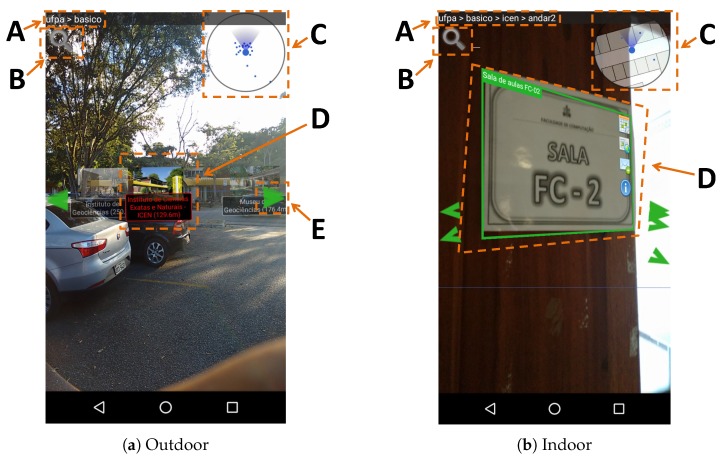
The graphical user interface of the prototype application:(**a**) the application running outdoors and following a route; and (**b**) the application running indoors and recognizing a room sign of a POI.

Figure 5a shows the main graphical interface of the application when the environment is outdoors. In “A”, the current user location is shown hierarchically. In “B” is the button responsible for searching POIs; the current version performs a simple search in the title, descriptions, and key-words of each place or object. In “C”, the mini radar shows the position of POIs around the user. In “D”, is the virtual marker of a POI; it is marked in red to indicate that this is the next step of the desired route. In “E”, some arrows are shown to indicate that there are more POIs in the pointed direction.

Figure 5b shows the changes that occur in the application when the user moves to an indoor environment. In “A”, the location is more detailed, situating the user on the second floor of the building. In “C”, the radar is adapted to show the floor plan of the building. In “D”, the virtual marker adapts to image recognition, which occurs when the user points their camera to some object that can be recognized and associated with a POI or IOI.

**Figure 6:**
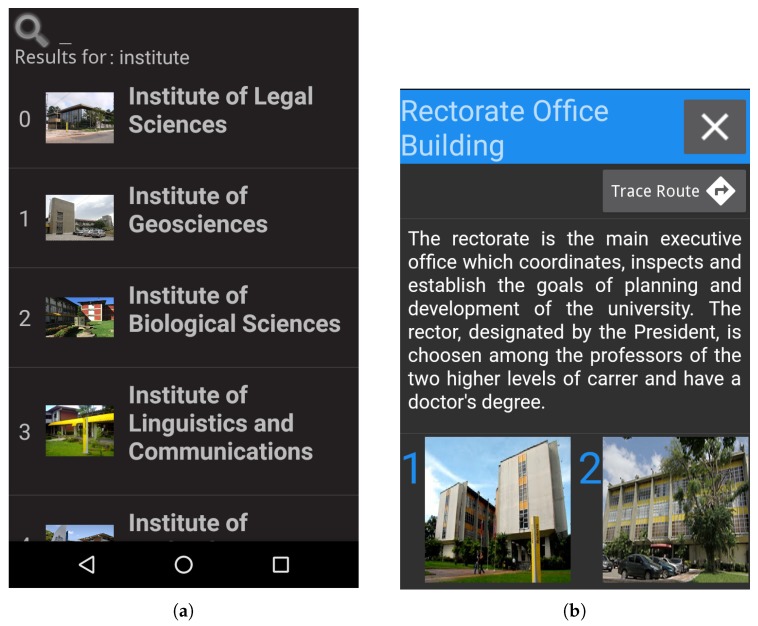
(**a**) The list of POIs returned by the search for the word “institute”, and (**b**) the displayed contents of a POI, where it is possible to read a text about the POI, see images and trace a route to the POI.

Figure 6a illustrates the list of POIs found by the search when the user enters a search term; in that case, the user entered the word “Institute”. Figure 6b shows the POI contents; in that case, the user can access a descriptive text, images of the POI and can trace a route to this POI.

### 4.3. AR Browser

The AR Browser helps the user to explore POIs in the neighborhood and guides the user to the POIs. The navigation feature contemplates actions in outdoors and indoors, and then changes from one environment to another.

**Figure 7:**
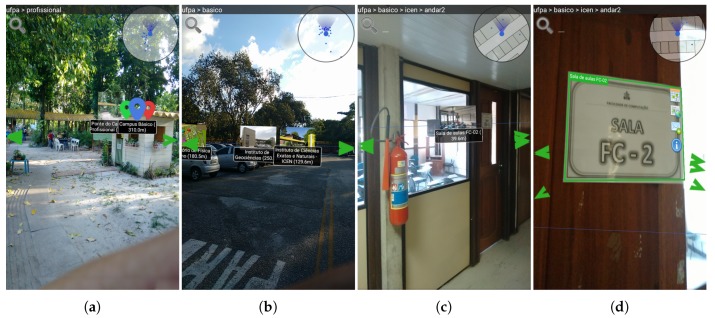
(**a**) The user is in the Professional sector and the application points to the Basic sector; (**b**) the user is in the Basic sector and the application points to the ICEN building; (**c**) the user is inside ICEN, and the application points to room FC-2; and (**d**) the user completed their trajectory, and the application recognizes the room sign.

Figure 7 shows four screenshots of the AR Browser functionality for different situations, and how the application adapts to such transitions. In this case, the user goes from the Professional sector to the room FC-2, in ICEN, which is in the Basic sector.

Figure 7a shows the application when the user is in the “Professional sector” AOI, thus the AOI marker represents all children of the Basic sector. Figure 7b shows when the user enters the “Basic sector” AOI: the POIs within it are revealed, including the ICEN marker. Once the user enters ICEN (Figure 7c), the indoor location is activated, positioning the IOIs relative to the user. In this way, the user is guided to the FC-2 room and can perform image recognition by pointing his camera at some pre-registered reference points (Figure 7d).

At any time during this whole trajectory, the user can obtain information about the POI/AOI by clicking on the respective virtual marker. Depending on the hierarchical location of the user, the application presents different POIs markers. The details of contents also change according to the user location; for example, when the user is outside a building, the application displays information about the locations and schedules of nearby events. However, when the user is facing a room, the system shows further details about the room itself, e.g., schedules of classes.

When the user wants more information about a POI visible on the screen of the mobile device, the user can select it by touching the POI virtual icon. When choosing a POI, the user can have access to the multimedia contents associated with it.

### 4.4. Indoor Localization Implementation

Since outdoor localization already has good accuracy for MAR applications, facing problems only when it is very close or inside the POIs, the application implements an indoor localization technique and a transition logic between AOIs and POIs environments.

There are several indoor localization strategies [31,32,33,34,35,36]. One method that deserves attention is Wi-Fi since indoor sites usually have it as part of a network infrastructure to provide Internet access. The method uses the signal strength of each access point to locate the user, eliminating the need to acquire new equipment.

Among the various localization strategies in the literature, the fingerprint method with Wi-Fi signals [37,38,39] was used in this prototype to validate the proposed model.

Any building containing scattered Access Points (APs) that emit signals (such as Wi-Fi) could have an indoor localization. The larger is the number of APs and the more scattered they are, the higher is the accuracy of localization. The indoor location in the ICEN building was implemented to conduct validation tests with the prototype, enabling the transition between localization through GPS and localization through Wi-Fi.

**Figure 8:**
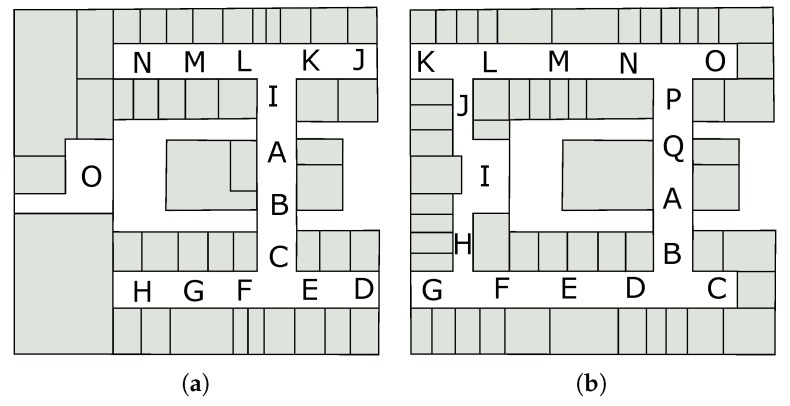
Floor plan of the two floors of the ICEN building with discrete locations (capital letters) used as fingerprint position.

The environment was mapped by collecting the intensity of the APs signals at predefined locations. Two hundred signal samples were collected from each point marked in Figure 8. Each sample is a vector of 55 dimensions, one for each AP in the ICEN building, containing the signal strength of the APs. The values in the dimensions vary between −120 (no signal) and −1 (highest intensity measured in decibels).

The AP intensity collection generates a dataset with a set of signals (the input vector) and the location of the user associated with those signals, including floor and position (the labels). These data were used to train Machine Learning (ML) models to generate a classifier: the input is the intensities that the mobile device is measuring, and the output is the location of the device. This approach is robust to more than one floor in the building, as vertical distance also affects AP signals.

Weka software [40] was used to perform the training and evaluation of the classifiers. To verify if the classification strategy is feasible, we tested training some classifiers that do not have many configurable parameters: KNN [41], Random Forest [42], Decision Tree [43], and Naive Bayes [44].

The ML model development led to a classifier committee: One classifier infers which floor the user is, and then other classifiers—one for each floor in the building—infer in which position the user is located on the associated floor. The same input vector was used to train the floor and location classifiers, with the difference being the labels. To train each location classifier on a given floor, only the signals collected from APs in the inferred floor was used as the input vector. Since the ICEN building has two floors, the resulting classifier committee contains three classifiers: one to infer the floor, one to infer position in the first floor, and one to infer position in the second floor.

Table 2 shows the metrics obtained by the Weka software when performing ten-fold cross-validation. The “Parameters” column shows the parameters used in training (Weka default values have been maintained). In all three cases, the Random Forest classifier performed better, even with a low number of random trees (20), thus we chose this classifier in the implementation of the application prototype.

After finalizing the training of the ML model, it was exported by Weka to be read inside the application. In total, 186 test measurements were performed using the application to verify if the inference is accurate in practice. These measurements obtained a hit rate of 83.33%.

### 4.5. Image Recognition Implementation

Image recognition should occur automatically, without the need to indicate which object should be recognized and when the process should start. Thus, to obtain the data used in the recognition process, the application uses the location information (AOIs, POIs, and IOIs near the user) and device orientation. This approach enables the application to have a conventional AR browsing at the same time that has the image recognition functionality running in parallel, as shown in Figure 7d.

**Figure 9:**
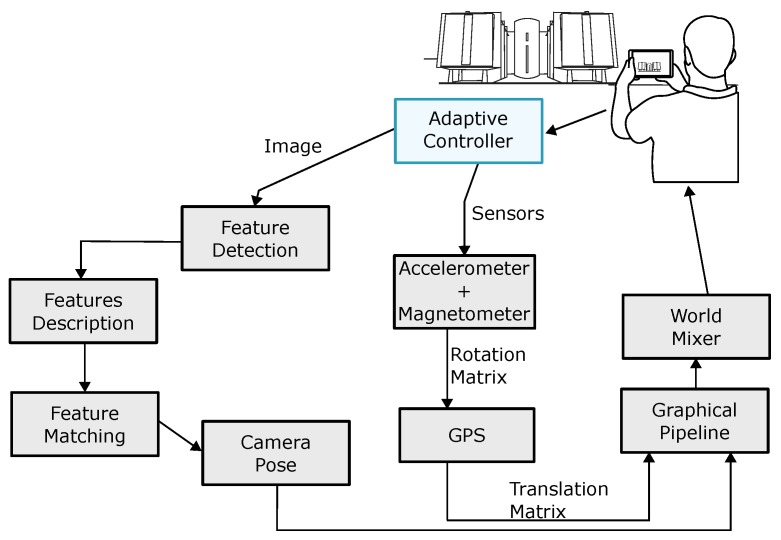
Hierarchical organization used in the validation prototype experiments.

Figure 9 shows the steps of the vision-based approach (indicated by the “Image” arrow), the sensor-based approach (indicated by the “Sensors” arrow), and the adaptive controller that chooses between each approach. For each frame obtained by the camera, the process is repeated to update the virtual information. The hybrid approach can combine image and sensors approaches in a variety of ways or can be used interleaved.

In a previous work [45], image recognition algorithms were tested to measure their efficacy in detecting certain characteristics. The ORB [46], BRISK [47], and AKAZE [48] algorithms were tested for detection and description of local features, and the algorithms RANSAC [49] and LMEDS [50] for the robust calculation of the camera pose.

The previous work [45] has shown that the system saves considerable time to perform the registration by sparsely using the ORB and using the sensors to complete the movement between each frame recognition. Although the registration time decreases as the algorithm stops using image recognition, the register suffers from alignment problems caused by the low accuracy of the sensors (e.g., the drift problem). Thus, the algorithm should balance the use of sensors and image recognition. Moreover, they conclude that sensors have low accuracy over short distances compared to computer vision techniques. The use of sensors in MAR applications is essential because it gives a precise location and orientation in scenarios that the user is far away from POIs and IOIs, and are faster than vision algorithms on average.

One of the contributions of the model presented in this article is the possibility of adapting the application to different user contexts, giving the flexibility of choosing the algorithm at runtime. This flexibility still allows the useful features of each algorithm to be combined in a better recognizer, as well as the use of a hybrid approach between these image recognizers and sensors. Works that measure the characteristics of each algorithm can help the model to decide how this combination can occur. For example, the model can decide to use the AKAZE for keyframes of video and the ORB for frames between the keyframe, since ORB is faster than AKAZE and AKAZE is more accurate than ORB.

### 4.6. Adaptive Logic

Algorithm 1 shows the pseudocode responsible for adapting the application when switching between environments. The code shows how the application can use the GPS to infer user position, and how it automatically activates the indoor localization when the user is inside a POI that has support for it. The algorithm locates the user recursively through the call to “locationChanged()” within the AOIs and POIs, and uses the appropriate location method through the “geo.obtainLocationBySensors()” function. At the end of the algorithm, the function “updateUserContent()” is called to adjust the application’s graphical interface and its contents according to the environment passed as parameter.

**Algorithm 1** Adapting the Interface According to the user location.
**function**locationChanged(context, newLocation)  ▹ Triggered when GPS position changes   listOfGeofences←context.getGeofences(newLocation);  **for each** geo **in** listOfGeofences **do**    **if** geo.contains(newLocation) **then**      **if** geo.isIndoor **then**               ▹ Change to short-range location         newLocation←geo.obtainLocationBySensors()      **end if**      locationChanged(geo,newLocation);          ▹ Recursive search       **break**;    **end if**  **end for**  **if** for loop does not find any Geofence **then**         ▹ The deepest geofence     updateUserContent(context);  **end if**
**end function**



Depending on the available content, various sensors can be enabled (or disabled to save battery) to make the augmented content available to the user. For example, in the implemented prototype, when the system can locate the contents of POI or nearby IOI by image recognition algorithms (i.e., the content already includes the proper markers to run an algorithm like ORB), the application can already use those markers to display a content overlay using augmented reality. Similarly, when the user is not near a POI or IOI that has markers to be recognized, the application automatically disables recognition algorithms to save resources such as the battery, processing, and memory.

The model does not set an optimal distance to disable or enable long-range or short-range sensors and features. However, these sensors are activated or deactivated according to the user’s hierarchical location, and to which places are closest to the user. For instance, if these nearby locations (considering the hierarchical position) have image-recognizable content, then the system automatically turns on the image recognizer; similarly, if a nearby location has properly registered Wi-Fi access points, then the system attempts to locate the user from those signals.

## 5. Prototype Evaluation

The evaluation test was performed with 11 volunteers using the prototype application in a real environment, following a predefined route. The route began in the Professional sector of UFPA (approximately 700 m from the target POI) and ended in the LABVIS room located on the second floor of the ICEN building. Note that, although we used the Basic sector as outdoor environment and the ICEN as an indoor environment in this evaluation, the model can handle sensor changes in other transitions such as indoor–indoor (e.g., two rooms in a building) and outdoor–outdoor (e.g., from a street to a park), depending of the available infrastructure the environments provide.

Initially, a pilot test was conducted with a participant to correct possible bugs in the application, and this pilot test was not considered for the analysis of the results.

The participants of the test had a background in computer science or computer engineering. Although the application is designed for any smartphone users interested in augmented reality, participants with knowledge in computer science or engineering were able to give more implementation-oriented feedback and bug fixes, which eventually occurred during testing. During the route, the participant was asked to put himself in the place of end-users. Tests with end-users of the application will be performed in further studies.

At the beginning of the test, the participant was asked to trace a route to LABVIS. Along the way, but without getting out of the path, the participant could stop at any point to explore and discover surrounding POIs through the application. Inside the ICEN building, participants were asked to perform the recognition of classroom plates to find information about places, such as the class schedule, e-mail list, and the schedule of extra activities.

The application recorded the participants’ routes during the experiment through text logs. Once the application records the user position, which may not be the correct position, one test conductor recorded the real user position using a manual application developed specifically for that purpose, providing a way to compare and evaluate the position measurements of the application.

### 5.1. Log Results

**Figure 10:**
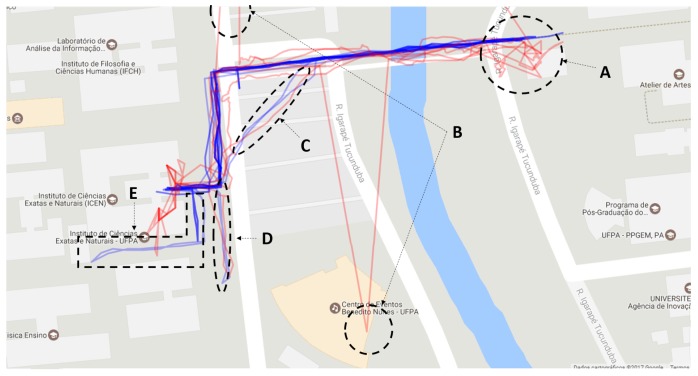
Overview of paths recorded by the application during the course (red lines) compared to the paths manually entered by an evaluator (blue line).

Figure 10 shows an overview of all participants’ paths. The blue lines show the course manually recorded by an evaluator and more accurately represent the path taken by the participant. The red lines represent the position registered by the application, which is the position used to register the virtual markers.

Figure 10 shows that there were two moments of critical failure in the positioning (B), which are the two red paths that are very distant from the blue path. The red lines show that although the positioning via GPS has good accuracy, it has low precision. This precision has more significant impacts on the register of the POI markers that are near to the user since this variability becomes more noticeable in short distances. In addition, the accuracy is not good at the beginning of the path (A) (right side of the bridge), possibly because the GPS delays to perform triangulation with the satellites that provide this service.

Two participants decided to cut their way through the parking lot (C), but the application properly recorded only one. Besides, two other participants realized that the marker was not pointing in the right direction of the target building when they got close to it and decided to follow the wrong path until the application corrected the problem (D and E).

**Figure 11:**
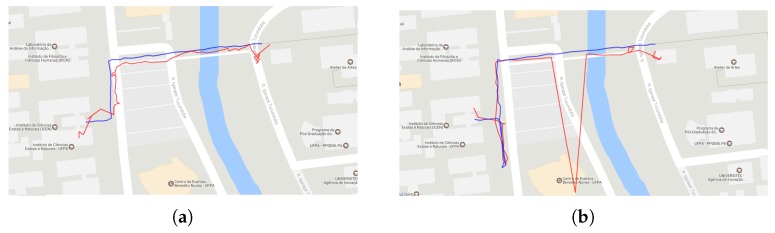
The routes taken by two participants: there is a variation of measurement near the target location (**a**); and an outlier in the GPS positioning (**b**).

Figure 11a highlights one route. The main problems of positioning occur at the beginning of the route and near the ICEN building (the target POI). Figure 11b shows problems in two moments: an outlier position when the participant is crossing the bridge, and near to the ICEN building because the application suggests that the building is further ahead.

When the participant enters the ICEN building, the application switches to the Wi-Fi localization and shows new virtual markers that indicate IOIs inside the building. The system also records the Wi-Fi location calculated through the power of the Wi-Fi signals for evaluation purposes. Moreover, the evaluator continued to record the participant’s paths in a separate application specially developed for this purpose.

**Figure 12:**
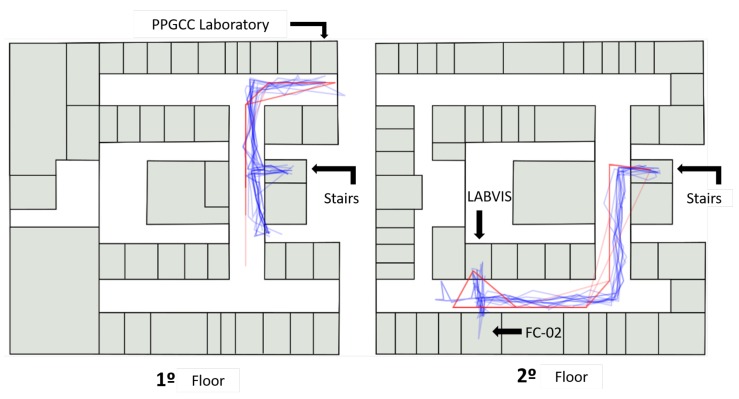
Overview of the path taken by the participants inside the ICEN building. The red lines represent the position obtained by Wi-Fi location, and an evaluator recorded the blue lines.

Figure 12 shows an overview of the path taken by the participants inside the ICEN building. On the first floor (left of Figure 12), participants were asked to find the post-graduate computer lab. After that, the participant was requested to locate the LABVIS room on the second floor.

**Figure 13:**
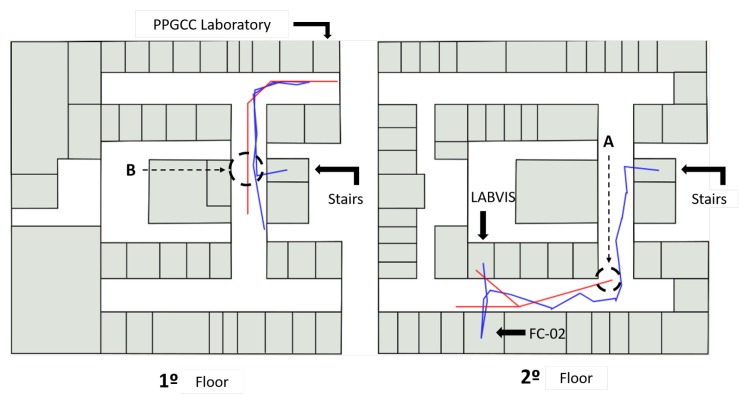
The highlight of a path from a participant inside the ICEN building: the location of the participant suddenly jump from (B) to (A).

Figure 13 highlights the trajectory of a participant and shows that, despite having good accuracy, the Wi-Fi positioning suffers from delays to update the position and this causes a time desynchronization between the actual location of the user and the one measured by the system. This case is highlighted in Figure 13, where the measured position jumps from position (B) on the first floor to the point (A) on the second floor.

**Figure 14:**
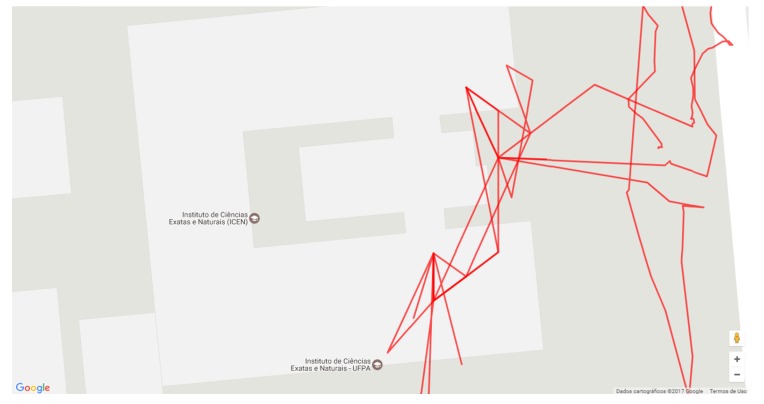
GPS based location when the participant is inside the ICEN. It is infeasible to use GPS in indoor environments with the tested GPS technology.

In this evaluation, the application continued to measure the GPS positioning even inside the building; note that this is only for purposes of comparison, and the application did indeed use only Wi-Fi sensors to perform registry when indoors. Figure 14 highlights the data and reveals that it would be impracticable to use GPS to perform the same task that was performed with the Wi-Fi location.

Thus, the adaptive model was crucial to make the user experience more fluid, as the application self-adapted automatically. The application changed the sensor used for registration from GPS to Wi-Fi to maintain a reasonable accuracy for the registry, without the user having to inform that he had entered in a different environment. As shown in Figure 14, if the application had continued to use GPS data in the indoor environment, the accuracy of the registry would be severely impaired.

### 5.2. Qualitative Results

This section describes the qualitative evaluation of the application considering some user tasks, such as POI awareness, self-location, POI marker perception, POI search, transition perception, content exploration, image recognition, route, search POI, and measure of distance. The evaluators defined those tasks based on common tasks in augmented reality applications, such as navigation, localization, and identification [51]. An interview with participants was also conducted after the test to collect data about the participant experience in using the application.

#### Procedure

The experiment employed three data collection methods: think-aloud protocol, observation, and interview [52]. The test conductor instructed participants at the beginning of the test to follow the think-aloud protocol so that participants could comment about their opinions, motivations, actions, and any other comments about the test experience. Besides that, the observer (test conductor) contributed by annotating problems that occurred during the tests (e.g., GPS failure), and by recording in audio the comments of participants.

After the test, the conductor interviewed the participants, asking questions to gather more directed information. The answers were also recorded in audio and used in the analysis. Table 3 shows the 12 questions used as a script to guide the interview. The interviewers did not influence the responses of the participants in any way, as they were limited to ask the questions present in the script and to clarify possible doubts.

To analyze the data gathered through interviews and think-aloud protocol, we employed an audio classification methodology [53]. The approach consists of a three-level codebook, where each level contains a range of possible categories that sentences can fit in. The audio was divided into text samples comprehending sentences that express a single idea of the participant. The text samples can be classified with up to three categories, one of each level. The classification process was conducted with three coders: each coder separately chose the categories they believed a sample fit into, and, if they did not agree, they discussed the classification until they reached a consensus. The coders first classified all the sentences in the first level of the codebook and then proceeded to levels two and three.

The first level of the codebook categorized the polarity of the comments (positive, negative, mixed, or neutral). If a sentence had mixed opinions, it was subdivided into its positive and negative components to be coded separately. After this first classification, only the negative sentences proceeded to the next levels of the codebook.

The second level categorized the task that the sample refers to. This level of the codebook was guided by the main tasks that users do in augmented reality applications (e.g., navigate, locate, and identify) [51]. Since these are low-level tasks, we used them to create a set of high-level tasks involved in the test (e.g., POI search and content exploration) that composes the categories of the second level.

Lastly, the third level categorized the problem that motivated the comment. Since all samples that reached the third level are negative ones, all of them had an underlying problem. The categories of this level were selected through observation during test conduction: the observer annotated any problem pointed by the user (e.g., GPS failure). After all the tests, the evaluators refined the list of problem categories, where similar issues were merged into a single category. After this aggregation, the final list of problems formed the third level of the codebook.

**Figure 15:**
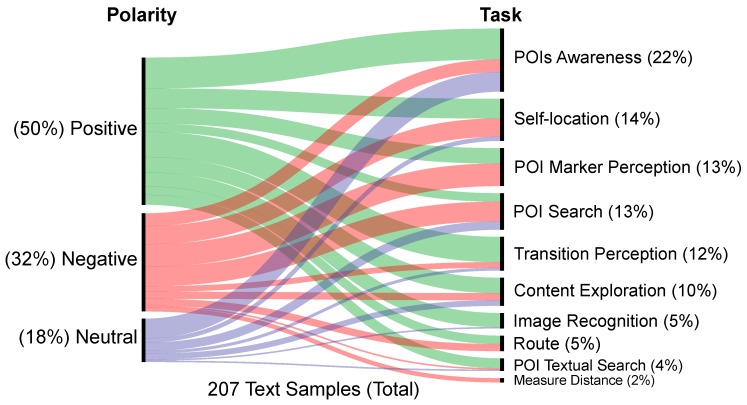
Correlation between text samples categories: polarity (left axis) and task (right axis).

Figure 15 shows a chart that correlates the classification of the first-level of the codebook (polarity, left axis) with the second-level (task, right axis). The length of each curve segment indicates the number of sentences that fit in that polarity/task pair.

Considering the primary goal of the proposed model, the transition between environments had a high proportion of positive comments. It is important to highlight that test conductors did not comment about the goal of the test with the participants. This result supports the idea that the model was efficient in fluid transitioning between environments.

**Figure 16:**
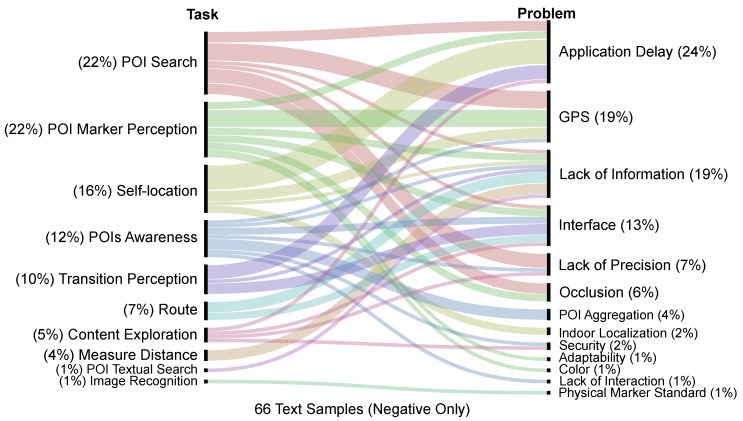
The correlation of tasks (left axis) and problems pointed by participants (right axis) in the negative comments.

The POI marker perception and POI search categories are the ones with a higher number of negative comments. The main cause of this high occurrence in both categories is the low accuracy or failure of GPS, which impacts the precision and delay of the system, causing markers to be positioned incorrectly on the screen. Figure 16 details the problems that motivated the negative comments.

The self-localization had problems mainly due to the delay in updating the application and to GPS failures. Other causes of self-localization issues include lack of information and indoors localization faults. Problems related to transition perception were also mainly due to the delay in updating the application position from outdoors to indoors, which impacts the interface update to reflect the transition (e.g., minimaps and route cues that take more time to update to indoors mode). This problem is partly due to the GPS accuracy in identifying when the participant had entered the indoors location. An approach that could mitigate this problem would be the use of dead reckoning to predict the approximate moment that the user transition between environments.

## 6. Conclusions and Future Work

This article presents an adaptive model for MAR applications to perform seamless transitions between environments, allowing similar user experiences in both indoor and outdoor environments using the same application. The process of adaptation is related to the change of the application in the following aspects:

- The dataset level, selecting and displaying only data that are near to the user;
- How to present virtual markers to the user;
- Interface adaptation, mainly in the aspect of the radar for the discovery of POIs;
- Use of image recognition techniques according to the position and orientation of the user;
- Automatic change of augmented reality tracking method; and
- The hybrid tracking choosing image recognition algorithms and its use alternately with sensors.

The architectural model of adaptive transitions between environments is situated as an additional step in the MAR application pipeline. The model presents the interleaved use of localization techniques from short-range and long-range sensors, and computer vision algorithms, associated with a hierarchy of geographical areas. The main novelty of the model is the fluid transition attained from using different localization techniques (such as GPS and Wi-Fi), the use of hierarchical location structures that contribute to the transition logic between environments in the model, and the automatic switching of the tracking method that improves the accuracy of registration and the consumption of resources, such as battery. In addition, a prototype was developed to carry out validation tests of the model.

The recorded GPS and Wi-Fi positioning showed that GPS has low accuracy over many uses, has low accuracy problems when the user is close to the target POI, and is infeasible to use as localization technique in an indoor environment given the restrictions of the current technology. Hence, adapting the positioning using Wi-Fi signals when the user enters a building reduces the problem, although there are still some challenges, such as delays in the update.

The participant feedback during the validation tests with the prototype pointed out that the adaptive measures implemented in the application were both perceived and evaluated positively, and that the application has a high potential for the discovery and exploration of new places, mainly for sectors such as tourism. The validation identified delay issues when updating the information, and confirmed problems with GPS accuracy when the user is near or within a POI, causing markers to be drawn incorrectly.

Adaptations of the graphical interface helped the user to self-locate more adequately in both indoor and outdoor environments. One of the clearest adaptations in the interface of the application was the mini-radar that shows the floor plan of the building when indoors. The perception of transition between environments presented a high percentage of positive comments, showing that the model was efficient in fluidly transitioning between environments.

Additionally, the route to a POI or IOI followed a step-by-step flow that made the test participants reach the desired location regardless of whether this place is outdoors or indoors. One participant even suggested that the route to the POI was even more personalized with messages such as “follow the corridor” and “the last door on the right” for indoor environments and “cross the bridge” and “turn right at next corner” for outdoor environments.

The implemented prototype makes extensive use of various sensors and processing features, which has an impact on the battery consumption of devices. One way to consume less battery power is to use sensors less often, using motion prediction algorithms to compensate. In practice, using only sensors does not make application usage impossible on the consumer market, but it indeed limits usage to devices that have proper processing and battery capacities, and also to ones that have available sensors.

Numerous future works can further investigate the presented model. For example, expansions in the model can also include information about the current time and climate of the region from a cloud service. Thus, when using an image recognition algorithm, the model can choose one more robust to low-light settings. Another possible expansion of the study is the use of more types of location sensors—such as Bluetooth Beacons and RFIDs—exchanging between them depending on the quality of the signal, or even using them simultaneously. Another further work is the improvement of image recognition techniques for indoor locations, such as the use of SLAM-based algorithms for environment mapping.

In addition, we have identified the research directions for future related works: perform a study to decrease the delay in displaying information to the user by doing predictive analysis of the user walk (dead reckoning), which can also be used to save battery; conduct studies with new indoor and outdoor tracking technologies; perform tests with other computer vision algorithms; attempt to optimize the algorithms involved in indoor and outdoor localization; investigate the use of crowd-sourcing to improve the accuracy of localization in indoor environments; and conduct studies about the scalability of the on-demand content available to the user location. For the later, future works can further study new approaches on how to retrieve information from a data service provider using the user context (e.g., localization, point of view, device characteristics), contributing to advance the literature in this related topic [54,55,56,57].

## Figures and Tables

**Table 1:** The overview of related works and its characteristics. The table shows the year, the proposed localization methods, and how the methods were combined. The abbreviations used in the table header mean: Head Mounted Display (HMD), Smartphone (Sm), Wi-Fi (WF), Bluetooth (Bl), Magnetic Field (MF), Global Positioning System (GPS), Natural Marker (NM), Fiducial Marker (FM), Combined Usage (CU), and Fluid Transition (FT).

		Devices		Short-Range Sensors			Long-Range Sensors	Image-Based Localization		Multimodal Localization	
Work	Year	HMD	Sm	WF	Bl	MF	GPS	NM	FM	CU	FT
[8]	2000	✔	✘	✘	✘	✘	✔	✘	✔	✔	✘
[9]	2003	✔	✘	✘	✘	✘	✔	✘	✔	✔	✘
[10]	2004	✔	✘	✘	✘	✘	✔	✘	✔	✔	✘
[11]	2007	✔	✘	✘	✘	✔	✘	✘	✔	✘	✘
[20]	2007	✔	✘	✘	✔	✘	✔	✘	✘	✔	✘
[21]	2009	✘	✔	✔	✘	✘	✘	✘	✘	✔	✘
[13]	2011	✘	✔	✘	✘	✘	✔	✘	✔	✔	✘
[14]	2013	✔	✘	✔	✔	✔	✔	✘	✘	✔	✘
[12]	2015	✘	✔	✘	✘	✘	✔	✘	✘	✘	✘
[15]	2015	✘	✔	✔	✘	✘	✔	✘	✘	✔	✘
[17]	2015	✘	✔	✘	✘	✘	✔	✔	✘	✔	✘
[16]	2016	✘	✔	✘	✘	✔	✔	✘	✘	✘	✔
[18]	2016	✘	✔	✘	✘	✘	✘	✔	✘	✘	✘
[19]	2019	✘	✔	✔	✘	✘	✘	✔	✘	✔	✘
Our Proposal	2019	✘	✔	✔	✘	✘	✔	✔	✘	✘	✔

**Table 2:** The Precision, Recall, and F-Measure of the location classifiers training. Best values in bold.

Type of Classification	Classifier Algorithm	Parameters	Precision	Recall	F-Measure
Floor	Random Forest	I = 20	**0.999**	**0.999**	**0.999**
	Decision Tree	C = 0.25	0.996	0.996	0.996
	KNN	K = 3	0.987	0.987	0.987
	Naive Bayes	-	0.738	0.688	0.680
First Floor Location	Random Forest	I = 20	**0.999**	**0.999**	**0.999**
	Decision Tree	C = 0.25	0.997	0.997	0.997
	KNN	K = 3	0.981	0.981	0.981
	Naive Bayes	-	0.917	0.900	0.899
Second Floor Location	Random Forest	I = 20	**0.999**	**0.999**	**0.999**
	Decision Tree	C = 0.25	0.996	0.996	0.996
	KNN	K = 3	0.990	0.990	0.990
	Naive Bayes	-	0.961	0.961	0.960

**Table 3:** The questions asked to the participants during the interview.

1	Did the application help you know where you were?
2	Did you get lost? If so, in which situation did this occur?
3	Do you think that virtual markers were positioned in the right place?
	Was there any difference in marker position when indoors and when outdoors?
4	Did you notice if there were changes in the application? Which ones? When did they happen?
5	Do you think that visual cues were related to real locations, or that they were floating on the screen unrelated to the environment?
6	What are the strengths and weaknesses of exploring content in this way?
7	Did the application help you in discovering items? Has it improved your perception?
8	Do you think the application has adapted to the environment you are in?
9	Did you consider image recognition useful for exploring content?
10	What were the strengths and weaknesses of the application in presenting information as you approach or enter places?
11	Do you prefer to have all the information available from the beginning, or gradually while approaching places?
12	In general, in what ways can the application improve?
